# Efficient Alignment of RNAs with Pseudoknots Using Sequence Alignment Constraints

**DOI:** 10.1155/2009/491074

**Published:** 2009-02-12

**Authors:** Byung-Jun Yoon

**Affiliations:** 1 Department of Electrical and Computer Engineering, Texas A&M University, College Station, TX 77843-3128, USA

## Abstract

When aligning RNAs, it is important to consider both the secondary structure similarity and primary sequence similarity to find an accurate alignment. However, algorithms that can handle RNA secondary structures typically have high computational complexity that limits their utility. For this reason, there have been a number of attempts to find useful alignment constraints that can reduce the computations without sacrificing the alignment accuracy. In this paper, we propose a new method for finding effective alignment constraints for fast and accurate structural alignment of RNAs, including pseudoknots. In the proposed method, we use a profile-HMM to identify the â€œseedâ€� regions that can be aligned with high confidence. We also estimate the position range of the aligned bases that are located outside the seed regions. The location of the seed regions and the estimated range of the alignment positions are then used to establish the sequence alignment constraints. We incorporated the proposed constraints into the profile context-sensitive HMM (profile-csHMM) based RNA structural alignment algorithm. Experiments indicate that the proposed method can make the alignment speed up to 11 times faster without degrading the accuracy of the RNA alignment.

## 1. Introduction

Sequence alignment lies at the heart of various computational methods that are used for analyzing biological sequences, such as RNAs and proteins. Alignment algorithms have been extensively used for comparing sequences to identify homologues, predict their structures, and infer their biological functions. Many functional noncoding RNAs (ncRNAs) are known to conserve their base paired secondary structure as well as their primary sequence [1]. For this reason, when aligning RNAs, it is important to consider both structure and sequence similarities in order to find an accurate alignment that is biologically meaningful. For a similar reason, it is expedient to employ scoring schemes that can reasonably combine contributions from the secondary structure similarity as well as primary sequence similarity when performing an RNA similarity search, as it can significantly reduce the number of false-positive predictions [2].

Conservation of the secondary structure gives rise to complicated symbol correlations between the pairing bases in the RNA sequence. Therefore, in order to take structural similarity into account, RNA alignment and search algorithms need to handle these base correlations in a principled manner. Until now, a number of probabilistic models have been proposed for this purpose [2, 3], where *stochastic context-free grammars (SCFGs)* and their variants have been especially popular. A typical problem of these models and the relevant algorithms is the high computational complexity. For example, the *Cocke-Younger-Kasami (CYK) algorithm* used in the SCFG-based alignment and search has a complexity of , where  is the length of the RNA that is to be aligned. Algorithms for simultaneous folding and alignment of RNAs (typically referred to as the *Sankoff algorithm*) have an even higher complexity, which require  computations for aligning  RNAs of length  [4]. The aforementioned algorithms do not consider pseudoknots, which are RNA secondary structures with crossing base-pairs, and there will be a steep increase in complexity if we begin to consider such pseudoknots. Pseudoknots are often ignored by many algorithms since they significantly increase the computational complexity.

The high computational cost of RNA alignment and search algorithms limits their utility in practical applications, especially when the RNA of interest is long. To cope with this problem, there have been extensive research efforts to develop heuristic methods that can make these algorithms faster without degrading the accuracy. For example, let us consider the simultaneous folding and alignment algorithm. Its computational complexity is already  for aligning just two RNAs, making it practically unusable for a larger number of RNAs. Even for pairwise alignments, the algorithm becomes quickly infeasible as the RNAs get longer. Therefore, in order to utilize these algorithms in practical applications, it is essential that we first reduce their computations. For this reason, most of the pairwise RNA alignment algorithms adopt various tricks to minimize the alignment time [5–11]. Similarly, a number of methods have been proposed to make RNA similarity searching faster, where the prescreening approach is a good example [12–15]. The prescreening approach uses a simple model, such as a *profile hidden Markov model (profile-HMM)*, to identify the regions that have a reasonable amount of (sequence) similarity. Only these regions will be passed to a more complex model, such as a *covariance model (CM; profile-SCFG)* [3] or a *profile context-sensitive HMM (profile-csHMM)* [16, 17], for further inspection. In fact, these are just a few examples, and there also exist other approaches for making RNA alignment and RNA search algorithms faster [18, 19].

Recently, we proposed an efficient RNA structural alignment algorithm based on profile-csHMMs, which can also be used for aligning RNAs that contain pseudoknots [17]. This algorithm finds the optimal alignment between a structured reference RNA and an unstructured target RNA, by taking both structure and sequence similarities into account. It was demonstrated that the profile-csHMM algorithm can find accurate alignment of RNA pseudoknots [17]. In this paper, we propose a novel method for finding effective sequence alignment constraints that can improve the computational efficiency of the profile-csHMM structural alignment algorithm. The overall organization of the paper is as follows. In Section 2, we describe the concept of constrained alignment and briefly review some of the existing methods for finding the alignment constraints. After the review, we propose a new method for estimating the alignment constraints in Section 3. Finally, Section 4 describes how these constraints can be incorporated into the profile-csHMM based structural alignment method. Experimental results will be presented at the end of Section 4, which demonstrate the effectiveness of the proposed approach.

## 2. Constrained Sequence Alignment

**Figure 1:**
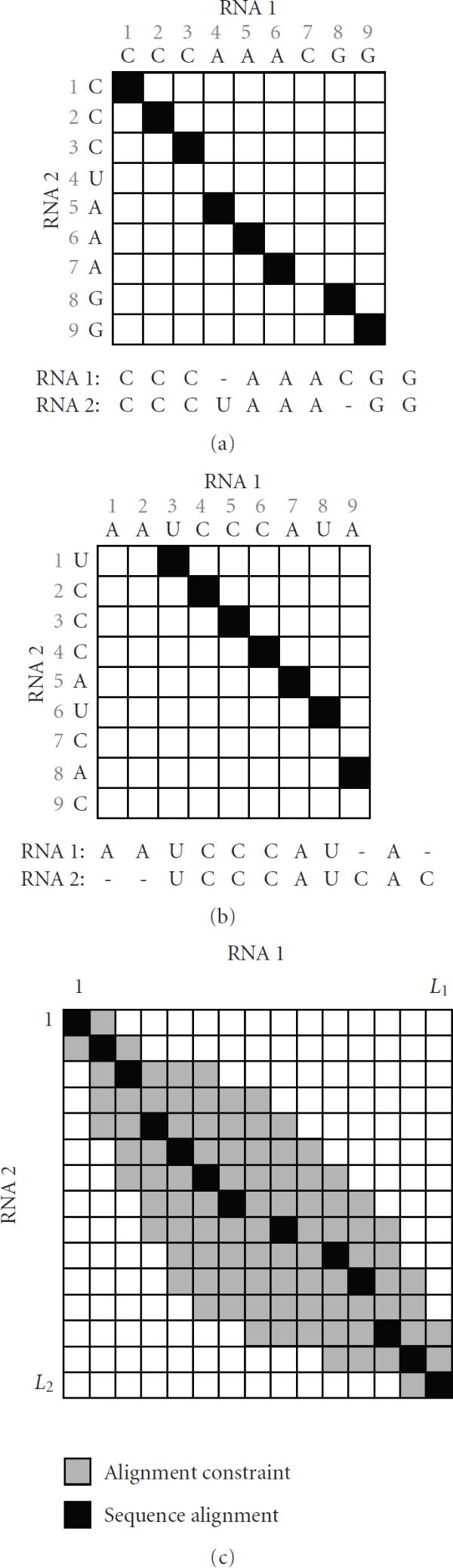
**(a), (b) Examples of sequence alignment**. The black squares denote the aligned positions. (c) Illustration of constrained alignment. The final sequence alignment (shown in black) should lie inside the constrained region (shown in gray).

Let us assume that we want to find the alignment of two RNAs  (RNA-1) and  (RNA-2). The predicted sequence alignment can be uniquely represented by the set of aligned bases  in a matrix. For example, let us consider the RNA alignment in Figure 1(a). The matrix shows the positions of the aligned bases, where a black square at  indicates that the bases  and  are aligned to each other. As we can see in Figure 1(a), the sequence alignment can be represented by a "path" of aligned base positions  in the alignment matrix. Another example is shown in Figure 1(b) for a slightly different alignment. Without any prior constraints, any base in one RNA can be aligned to any base in the other RNA, hence the "alignment path" can be located anywhere in the matrix.

Now, assume that we are given some prior information about the region where the aligned bases should be located. This is illustrated in Figure 1(c), where the gray area depicts the possible position  of the aligned bases  and . Knowing that all the aligned bases should be located inside the given region, we only have to consider the alignment paths that are contained in this region when finding the RNA alignment. Consequently, the search space for finding the optimal alignment is reduced, bringing down the overall computational cost. The reduction in time achieved by restricting the alignment space is typically much larger compared to the reduction in the alignment space itself, since the computational complexity of most RNA alignment algorithms is a high-order polynomial of the RNA length. It has to be noted that the alignment accuracy will not be affected as far as the optimal alignment (the black path in Figure 1(c)) is contained in the constraining region (the gray area in Figure 1(c)).

As illustrated in the previous example, using appropriate sequence alignment constraints can greatly enhance the efficiency of the alignment algorithm. So, the natural question is how we can predict good alignment constraints that can minimize the alignment time without degrading the alignment accuracy. Until now, various methods have been proposed for restricting the alignment space to improve the efficiency of diverse RNA alignment and search algorithms [5–9, 11, 17, 18]. For example, the query-dependent banding (QDB) method [18] is used to make CM-based RNA alignment algorithms faster, by excluding the regions in the dynamic programming matrix that have insignificant probability. These regions can be precomputed based on the given CM and do not depend on the target database. *Foldalign* [7], an algorithm for simultaneous RNA folding and alignment, limits the maximum length of the RNA-motif as well as the maximum length difference between the subsequences that are being compared. Recent implementation of Foldalign [8] adopts a heuristic that prunes the dynamic programming matrix in order to reduce the overall time and memory requirements. Another RNA alignment and structure prediction algorithm called *Stemloc* [9] constrains the solution space by using "fold envelopes" and "alignment envelopes." The fold envelopes are used to restrict the search over secondary structures and the alignment envelopes are used to restrict the possible alignments between the given sequences.

**Figure 2:**
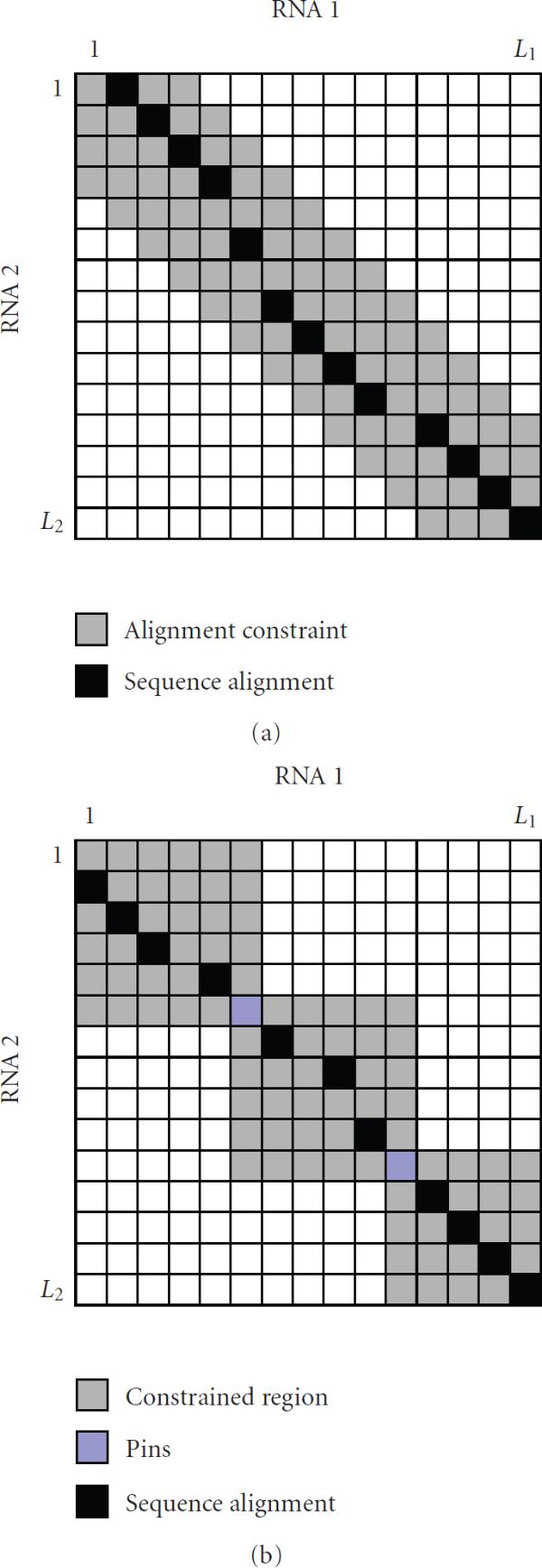
**(a) Alignment constraint in Dynalign **[11]. The maximum distance between the aligned bases is restricted. (b) Consan [5] constrains the alignment space by fixing the "pins," or confidently aligned base positions.

A recent implementation of *Dynalign* [11], a joint alignment and secondary structure prediction algorithm for two RNAs, assumes that the aligned bases in the respective RNAs should be located within a certain distance. To be more precise, the th base  in RNA-1 () can be aligned to the th base  in RNA-2 (), only if the following condition is satisfied: (1)

for a given . For convenience, we refer to this constraint as the *M-constraint*. The parameter  is used to specify the maximum distance between the alignable bases. By imposing this constraint, we are restricting the number of insertions and deletions in homologous sequences, which is a reasonable assumption for real biological sequences. The constrained alignment space is band-shaped as depicted in Figure 2(a). Despite its simplicity, is has been shown that the proposed constraint works reasonably well [6, 11]. The latest implementation of Dynalign [6] takes a more principled approach for estimating the alignment region. In the new approach, a hidden Markov model (HMM) is used to estimate the set  of aligned positions , whose *co-incidence probability* is larger than a reasonably low threshold : (2)

Two bases  and  are said to be coincident if (i) they are either aligned to each other, or (ii) if  is inserted in the region that immediately follows  which is aligned to , or vice versa [6]. The estimated set  is used to constrain the final alignment. It was demonstrated that this technique can provide significant savings in computational time as well as a small improvement in alignment accuracy [6]. Another pairwise folding and alignment algorithm called *Consan* [5] first finds the confidently aligned base positions, referred to as "pins," and constrains the RNA alignment by fixing these positions. This is illustrated in Figure 2(b). The set of pins  is estimated using a pair-HMM, by looking for base positions  whose alignment probability  exceeds some threshold , which is close to unity. This set  can be written as follows: (3)

For every predicted pin , the bases  and  are forced to be aligned to each other in the final alignment. While Dynalign [6] finds the set of *all possible* alignment positions, Consan [5] tries to find only a small set of alignment positions that *must* be included in the final alignment.

Although the previous alignment constraints [5, 6, 11] were mainly used to speed up Sankoff-style joint alignment and folding algorithms, similar ideas can be used to expedite dynamic programming alignment algorithms such as the CYK algorithm [3] for CMs and the *sequential component adjoining (SCA) algorithm* [16, 17] for profile-csHMMs. In the following section, we propose a new method for finding effective sequence alignment constraints that are especially useful for making these algorithms faster.

## 3. Alignment Constraints for RNA Family-Specific Models

Let us assume that we have a reference RNA whose structure is known. This can be either the consensus sequence of an RNA family or simply a single RNA sequence. Also assume that we are given a target RNA with an unknown structure, which might be a putative member of the same family. We want to find the optimal alignment between these RNAs by considering both their sequence and structural similarities. This *structural alignment* can be used for predicting the secondary structure of a new homologue [17, 20] or performing an RNA similarity search to identify new members in the same RNA family [3]. In order to find the structural alignment, we first construct a stochastic model (such as a profile-csHMM or a CM) that can closely represent the reference RNA. Then we use a dynamic programming alignment algorithm to find the best alignment between the reference RNA (represented by the constructed model) and the target RNA. Although the computational complexity of these algorithms is generally lower than that of Sankoff-style algorithms, it still ranges between  and  for a target RNA of length . For RNAs without pseudoknots, the computational complexity of the alignment algorithm will be . The complexity for aligning pseudoknotted RNAs is at least , and it can become higher as the structure gets more complex. This renders the dynamic programming algorithms impractical for aligning long RNAs or scanning a large database, and using effective alignment constraints can be greatly helpful in relieving this problem.

### 3.1. Motivation for Estimating Constraints Based on Predicted Alignment Positions

When we are interested in a specific RNA family, it will be more appropriate to establish the alignment constraints based on the member sequences in the given family. Therefore, it will be more desirable to use a *family-specific* model for finding the constraints, rather than using a *general* model that applies to all RNAs as in [5, 6]. However, in many practical situations, we may not have enough number of sequences in the given family for reliably estimating the model parameters. As we can see in (2) and (3), the alignment constraints in Dynalign [6] and Consan [5] strongly depend on the estimated alignment probabilities. Although the alignment constraints used in these methods are expected to work well when we have a large number of training sequences, they are not suitable when only a handful of RNAs are available for training the model.

**Figure 3:**
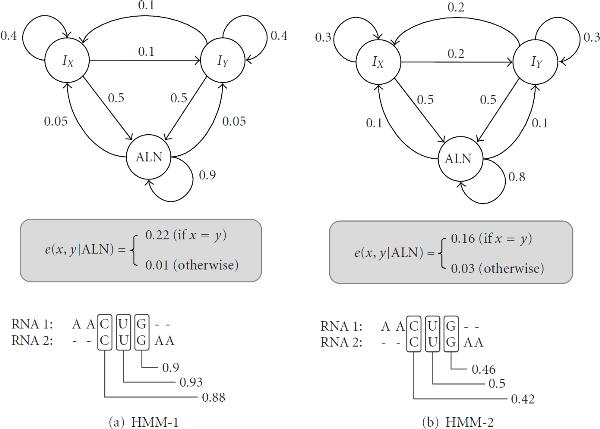
**Two pair-HMMs with slightly different parameters**. Both pair-HMMs have three states, , , and , which represent base alignment, base insertion in RNA-1, and base insertion in RNA-2, respectively.HMM-1HMM-2

So, how can we find efficient alignment constraints for a family-specific model when we have only a limited number of sequences in the reference RNA family? In order to answer this question, let us consider the pair-HMMs shown in Figure 3. Both pair-HMMs have three hidden states, , , and , for base alignment, base insertion in  (RNA-1), and base insertion in  (RNA-2), respectively. The state  emits a pair of aligned bases  and . The insert state  emits an unaligned base  in , and similarly, the state  emits an unaligned base  in . These pair-HMMs can be used for finding a *sequence-based alignment* between two RNAs, and for estimating the base alignment probabilities. It is called a *sequence-based alignment*, since the alignment is obtained based on sequence similarity alone. Similar models have been used to find the alignment constraints (2) and (3) in Dynalign [6] and Consan [5], respectively. In this example, the transition probabilities of the pair-HMMs are shown along the arrows. We assume that the probability of entering a state in the beginning is identical for all three states. The emission probability  of a pair of aligned bases  is shown inside the box below the respective HMMs in Figure 3. Finally, the emission probability at an insert state is specified as follows:(4)

Now, let us assume that we want to find the sequence-based alignment of the following RNAs:(5)

using the pair-HMM shown in Figure 3(a). Using the Viterbi algorithm, we can get the following alignment(6)

For each aligned pair , we can compute the alignment probability  using the *forward-backward algorithm* [21]. The estimated base alignment probabilities are shown in Figure 3(a), below the RNA alignment. The estimated alignment probabilities are close to unity, indicating that we can be more or less confident about the predicted base alignments. Now, let us repeat this process using the pair-HMM shown in Figure 3(b), which has slightly different parameters. As we can see in Figure 3(b), HMM-2 finds the same alignment as HMM-1, but the estimated alignment probabilities are significantly different from the previous estimates. This example clearly shows that the estimation of the base alignment probability  can be very sensitive to small changes in the model parameters. This implies that the alignment constraint in (3), which depends on , may not be reliable when we do not have enough training data to accurately estimate the HMM parameters. Compared to this, the alignment constraint in (2) might be more reliable, as the coincidence probability  used to estimate the constraint also includes the insertion probabilities. However, the predicted constraint will nevertheless depend on the parameters of the HMM to a considerable extent.

However, one thing we can notice by comparing Figures 3(a) and 3(b) is that, despite the large difference in the estimated alignment probabilities, the resulting sequence alignments are identical. In fact, the alignment positions in an optimal sequence alignment are not very sensitive to small parameter changes, and as a result, HMMs with reasonably similar parameters often yield almost identical alignment results. This motivates us to exploit the *aligned base positions* for establishing the alignment constraints, instead of using the *base alignment probabilities*.

### 3.2. Finding Seed Regions Using a Profile-HMM

Based on the previous observation, we propose a new method that utilizes the predicted alignment positions to find the alignment constraints. As before, let us denote the structured reference RNA as  (RNA-1) and the unstructured target RNA as  (RNA-2). Ultimately, we want to find the *structural alignment* of these RNAs. However, since the dynamic programming algorithm for finding the structural alignment is computationally expensive, we want to come up with effective alignment constraints that can speed up the alignment.

For this purpose, we first build a profile-HMM [3] based on the reference RNA family. This model is used to find the *sequence-based alignment* between the reference RNA (represented by the profile-HMM) and the target RNA. Secondly, we identify the regions that consist of multiple consecutive base alignments, or base matches. Although a single base match may not be meaningful by itself, having a region of consecutive matches often indicates that the alignment in the given region is reasonably accurate. This is especially true for those matches that are located in the middle of the region. For example, we can see in both Figures 3(a) and 3(b) that the alignment probability  is the largest for the alignment between  and , which is located between the matches  and . Therefore, we exclude the matches near the end and keep the remainder to obtain a set of reliable base alignments. The set of reliable contiguous matches is referred to as the *seed region*. The procedure for finding the seed regions can be summarized as follows.

(1) Find a sequence-based alignment between the RNAs.

(2) Identify all regions, and longest such regions, that consist of consecutive matches. Let  be the number of consecutive matches in a given region. Keep only those regions with .

(3) In each region, exclude the first  matches in the left end and the last  matches in the right end.

(4) The region that consists of the  remaining matches is defined as a seed region.

**Figure 4:**
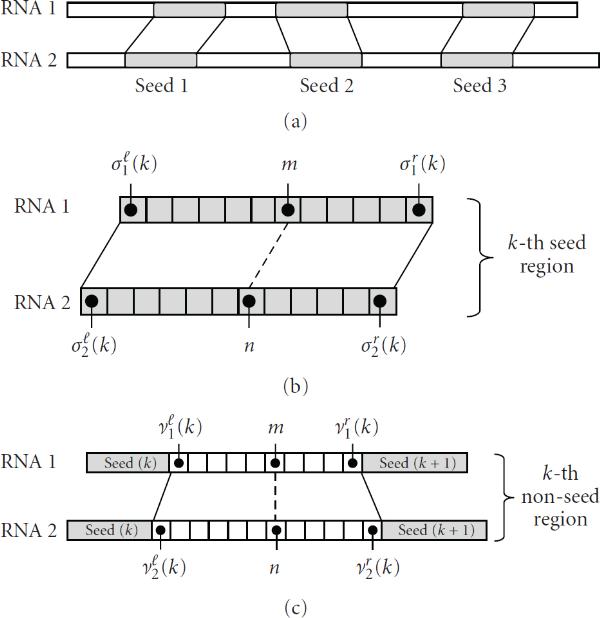
**Illustration of the proposed method**. (a) Seed regions are identified from the sequence-based alignment. (b) Example of a seed region, which consists of consecutive base matches. (c) Example of a nonseed region.

The integer parameters  and  define the seed regions during this process. In general, using a larger  will identify a smaller number of seed regions, and a larger  makes the seed regions contain fewer but more reliable base matches. Figure 4(a) illustrates an example alignment with three seed regions.

### 3.3. Constraints in a Seed Region

Assume that we have identified  seed regions according to the procedure described in Section 3.2. Since the base alignments in these regions are relatively reliable, we keep the alignment space in these regions small. Let us consider the th seed region illustrated in Figure 4(b). We denote the begin index and the end index of the th seed in RNA-1 as  and , respectively. The superscripts  and  in  and  stand for "left" and "right," respectively. Similarly, the begin and the end indices of the th seed in RNA-2 are denoted as  and , respectively. Since a seed region consists of consecutive base matches, we have(7)

where  is the length of the th seed. For convenience, we define  as the position difference between the aligned bases in the th seed(8)

Based on the th seed, we define the set of allowed alignment positions  as follows:(9)

For a base  that is contained in the th seed region of RNA-1, the parameter  restricts the distance between the base  (), to which  is aligned in the sequence-based alignment, and the base , to which  will be aligned in the final structural alignment. As the base alignments in the seed regions are reliable,  can be typically set to a small number. We find the set  for all , and these sets will be combined later to establish the final alignment constraints.

### 3.4. Constraints in a Nonseed Region

The predicted base alignments in the nonseed regions are generally less reliable compared to those inside the seed regions. Therefore, we define different alignment constraints for the bases contained in the nonseed regions, and make the constraints less stringent compared to (9). Let us consider the th nonseed region illustrated in Figure 4(c). The begin and end indices of the th nonseed region in RNA-1 are denoted by  and , respectively. Similarly,  and , respectively denote the begin and end indices of the corresponding nonseed region in RNA-2. Now, we define the set , which contains (i) all aligned base positions  in the th nonseed region, as well as (ii) the first and last positions  and  in this region:(10)

In practice, it is possible that there may be no aligned bases  in the given nonseed region. Including the terminal positions  and  of the th nonseed region in  ensures that the set  will never be empty. For the position pairs , we estimate the range of the position difference  as follows:(11)

Based on these values, we define the following set:(12)

which contains the alignable base positions  in the th nonseed region. Note that we use the same  in (9) and (12). Therefore, a larger  will relax the alignment constraints for both seed and nonseed regions, and a smaller  will make both constraints more stringent.

### 3.5. Overall Alignment Constraints

In Sections 3.3 and 3.4, we defined the alignment constraints in the seed regions as well as the constraints in the nonseed regions. Finally, we combine (9) and (12) to obtain the overall alignment constraints  as follows:(13)

This set  can be used to constrain the alignment space of the dynamic programming algorithm for finding the structural alignment of the given RNAs. When finding the RNA alignment, we allow a base  in RNA-1 to be aligned to a base  in RNA-2 only if the pair  is included in this set .

## 4. Experimental Results

To demonstrate the effectiveness of the proposed method, we applied the new alignment constraints to the profile-csHMM-based structural alignment method [17]. In the following, we provide a brief explanation about the experimental set-up and present the experimental results.

### 4.1. Profile-csHMM-Based Structural Alignment

**Figure 5:**
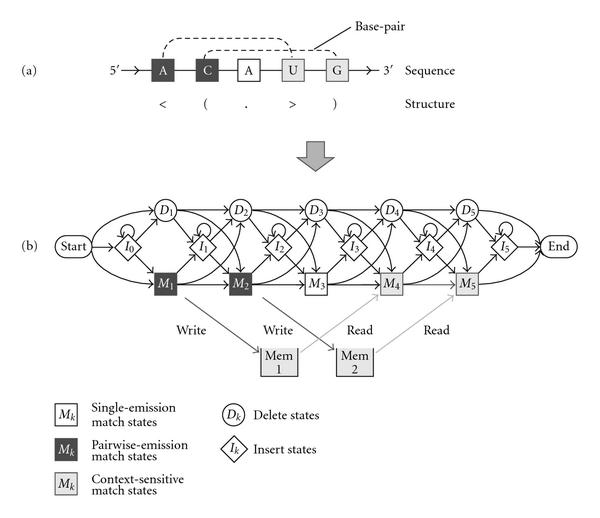
**Constructing a profile-csHMM**. (a) A reference RNA sequence with a known secondary structure. (b) The profile-csHMM that represents the given reference RNA.

Profile-csHMMs are a subclass of context-sensitive HMMs [22] that are especially useful for representing RNA sequence profiles and their secondary structure. In principle, profile-csHMMs can represent RNA secondary structures with any kind of base pairs [16, 17]. As a result, profile-csHMMs can also be used for aligning and predicting the structure of RNAs that contain pseudoknots, which cannot be done using the widely used SCFGs (or CMs). The profile-csHMM-based structural alignment algorithm proposed in [17] proceeds as follows. In the first place, a profile-csHMM is constructed based on a reference RNA sequence with a known structure. In [17], a single reference RNA was used to build the model. This can be used for performing a single RNA homology search, similar to the CM-based search proposed in [23]. Figure 5 illustrates an example, where a profile-csHMM is constructed based on a reference RNA that has two crossing base pairs. Obviously, we do not have enough training sequences to accurately estimate the model parameters in this case, hence the parameters of the profile-csHMM are chosen according to the scoring scheme proposed by Gorodkin et al. [24]. These scores can be viewed as normalized log-probabilities for observing base substitutions or gaps (insertions and deletions) in homologous RNAs. They have been used in a number of RNA alignment algorithms [20, 24], yielding accurate alignment results. The constructed profile-csHMM can then be used for finding the optimal structural alignment between the reference RNA and an unstructured target RNA, computing their alignment score, and predicting the secondary structure of the target RNA. A dynamic programming alignment algorithm called the *sequential component adjoining (SCA) algorithm* can be used for this purpose.

### 4.2. Estimating the Alignment Constraints

In order to estimate the alignment constraints for expediting the profile-csHMM alignment algorithm (or the SCA algorithm), we construct a profile-HMM-based on the same reference RNA. Note that unlike the profile-csHMM, the traditional profile-HMM reflects only the sequence characteristics of the reference RNA. Similar to the parameterization of the profile-csHMM described in Section 4.1, the parameters of the profile-HMM are also specified according to the scores in [24]. The resulting profile-HMM is used to estimate the sequence alignment constraints as we elaborated in Section 3. We use the estimated constraints to restrict the alignment space of the structural RNA alignment to reduce the overall computational load.

### 4.3. Choosing the Parameters for Constraint Estimation

Now, one practical question is how we should choose the parameters , , and  that are used to estimate the alignment constraints in Sections 3.3 and 3.4. Ideally, the predicted alignment constraints should minimize the alignment space without affecting the quality of the final structural alignment. Since the alignment constraints proposed in Section 3 are derived from the predicted seed regions, the alignment accuracy in these regions has a crucial impact on the accuracy of the proposed approach. For this reason, we estimated the average base alignment accuracy in the seed regions for the 5S rRNA and tRNA families in the Rfam database (version 8.1) [25]. We used the RNAs in the *seed alignment* of the respective family, as they have a relatively reliable secondary structure annotation. For each RNA family, we first chose a reference RNA among its members, and constructed a profile-HMM based on the chosen RNA. Then we aligned the remaining members to the reference RNA using the profile-HMM. For every sequence alignment, the predicted alignment positions have been compared to the correct positions in the database to estimate the alignment error rate. In order to get a reliable estimate, we repeated these experiments by using every member as the reference RNA. This resulted in 1, 182, 656 pairwise alignments for tRNAs and 345, 156 alignments for 5S rRNAs.

**Figure 6:**
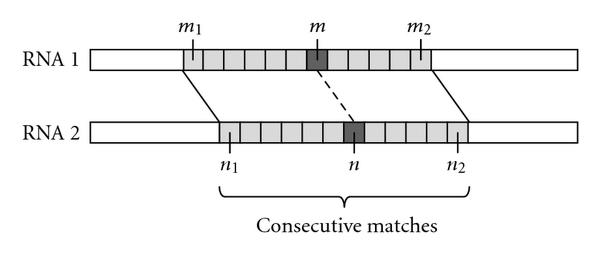
**A region that consists of consecutive base matches**.

Let us assume that the profile-HMM predicted that  in the reference RNA should be aligned to  in the target RNA. We want to estimate the probability of error for this prediction as a function of the following parameters:

(1) **: the number of consecutive base matches in the region containing the alignment ,

(2) **: the minimum distance between the given base alignment  and the terminal alignment positions  and . See Figure 6 for illustration.

Consider the example illustrated in Figure 6. The number of consecutive base matches, or the *length*, of the region containing  is(14)

and the minimum distance  is defined as(15)

Based on these definitions, we define(16)

This is the probability that the predicted base alignment  between  and  will be incorrect, given that the following hold:

(1) the length  of the alignment region containing  is at least ,

(2) there are at least  matches in the left-hand side of  as well as in the right-hand side.

**Figure 7:**
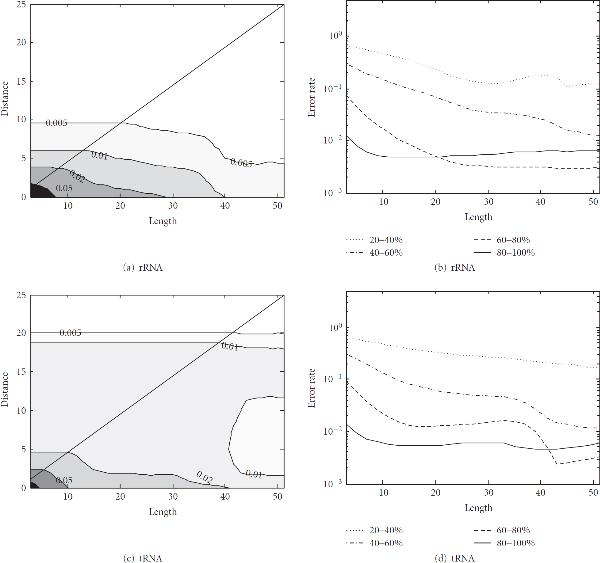
**Alignment error probability  in the seed regions**. (a) Contour plot of  for 5S rRNAs. Darker shade corresponds to higher  and lighter shade corresponds to lower . The misalignment probability  on the level curves are shown on top of the contours. (b) Misalignment probability for 5S rRNAs with different percentage identities. (c) Contour plot of  for tRNAs. (d) Misalignment probability for tRNAs with different percentage identities.rRNArRNAtRNAtRNA

Figure 7(a) shows the contour plot of the misalignment probability  for 5S rRNAs, where the -axis is for  and the -axis is for . On top of each contour curve, we show the corresponding misalignment probability  for the points  on the given curve. Darker shaded regions correspond to higher  and lighter shaded regions correspond to lower . The diagonal line representing  is shown in the plot as a reference. Note that, by definition, we have . Therefore, for any  such that , which corresponds to the region above the diagonal line, we will have .

As we would expect, the misalignment probability  becomes smaller as  and  get larger. Figure 7(b) shows the misalignment probability  of 5S rRNAs for . The pairwise alignments have been divided into different groups based on the *percentage identity* (or *percent sequence similarity*) of the aligned RNAs, and the alignment error probability  has been computed for the respective groups. As we can see in Figure 7(b), the error probability is generally lower for RNAs with higher percentage identity. This is expected, since the seed regions are predicted from a sequence-based alignment, which will be more accurate if the RNAs have higher sequence similarity. Figures 7(c) and 7(d) show the misalignment probability  for tRNAs, which have similar trends.

We also computed  for RNAs with  identity for different values of . This is summarized in Table 1. For example, the alignment error probability  for 5S rRNAs is 1.81% for  and 0.79% for . This implies that if we choose  and  when finding the seed regions (see Section 3.2), more than 98% of the alignments in the predicted seed regions will be correct. In our experiments, we observed that the misaligned bases were typically located within  base positions from the correct ones. This implies that if we let  or , most of the correct base alignments  will be included in the constrained alignment space  in (9). Therefore, imposing these constraints will not degrade the accuracy of the final structural alignment.

**Table 1:** Misalignment probability in the seed regions of RNAs with 60%  100% identity. The probability  has been estimated for five different values of .

	
	(7,3)	(9, 4)	(11, 5)	(13, 6)	(15, 7)
	Error (%)	Error (%)	Error (%)	Error (%)	Error (%)

5S ribosomal RNA	2.64	1.81	1.32	1.00	0.79
Transfer RNA	3.60	2.46	1.80	1.44	1.28

### 4.4. RNA Structural Alignment with the Proposed Constraints

As mentioned earlier, we applied the proposed constraints to the profile-csHMM-based structural alignment algorithm [17]. For our experiments, we chose six RNA families from the Rfam database [25]. The Flavi_pk3 RNAs were obtained from Rfam 7.0, which are now part of the Flavi_CRE family in Rfam 8.1. For other families, we obtained the RNAs from Rfam 8.1. Among the six families, two families, tRNAs and 5S rRNAs, do not contain pseudoknots in their secondary structures, while the other four families, Corona_pk3, HDV_ribozyme, Tombus_3IV, and Flavi_pk3, contain pseudoknots. The basic properties of these RNA families, such as the number of RNAs in the Rfam seed alignment, the average length of the member sequences, and their average percentage (sequence) identity, are shown in Table 2. For each RNA family, we performed the following experiment.

**Table 2:** Basic properties of the RNA families used in the experiments

	Number of seed sequences	Average length	Average percentage identity
Transfer RNA	1088	72.7	45
5S ribosomal RNA	602	116.8	61
Corona_pk3	14	62.5	70
HDV_ribozyme	15	88.8	95
Tombus_3_IV	18	64.5	94
Flavi_pk3	14	95.4	69

(1) Choose a reference RNA from the seed alignment.

(2) Construct a profile-HMM and a profile-csHMM-based on the reference RNA.

(3) Choose a different target RNA from the seed alignment.

(4) Estimate the alignment constraint using the profile-HMM.

(5) Apply the alignment constraint and find the structural alignment using the profile-csHMM.

(6) Repeat step-3 to step-5 for different target RNAs.

(7) Repeat step-1 to step-6 for different reference RNAs.

In order to measure the quality of the structural alignment, we predicted the secondary structure of the target RNA based on the structural alignment, and compared it to the trusted structure in the Rfam database. Then we counted the number of correctly predicted base pairs (TP; true-positives), the number of incorrectly predicted base pairs (FP; false-positives), and the number of true base pairs that could not be predicted (FN; false-negatives). Based on these numbers, we estimated the *sensitivity (SN)* and the *positive predictive value (PPV)* as follows:(17)

The sensitivity is defined as the fraction of base pairs in the trusted structure that could be predicted by the algorithm, and the positive predictive value is defined as the fraction of predicted base pairs that were correct.

We first tested the performance of the proposed approach on RNA families that do not contain pseudoknots. In order to compare the effectiveness of different alignment constraints, we repeated the above experiment for the following methods.

(1) Profile-csHMM + proposed alignment constraint (referred to as "PROPOSED").

(2) Profile-csHMM + *M*-constraint (referred to as "*M*-CONSTRAINT").

(3) Profile-csHMM (original implementation in [17]; referred to as "ORIGINAL").

Table 3 summarizes the average sensitivity (SN), positive predictive value (PPV), and alignment time for using different alignment constraints with the profile-csHMM-based structural alignment method. The CPU time for finding the alignment has been measured on a MacPro with two 2.8 GHz quad-core Intel Xeon processors and 4 GB memory. These results have been obtained from one thousand structural alignments of distinct pairs of RNAs that were chosen from the seed alignment of the respective RNA families. For these experiments, we used  for tRNAs and  for 5S rRNAs. These parameters were chosen based on the analysis in Section 4.3. In general, there will be a tradeoff between alignment accuracy and runtime. These parameters have been used as they provide a good balance between these two measures. Further discussion on this tradeoff can be found at the end of Section 4.4. For the *M*-constraint defined in (1), we used  as in [6]. As we can see in Table 3, all three methods were able to achieve accurate alignment results that were comparable to each other. However, adopting the proposed alignment constraint improved the average alignment speed significantly, which was around 7  68 times faster compared to the fixed *M*-constraint, and up to 2.4 times faster compared to the original implementation in [17] that uses a simple heuristic.

**Table 3:** Average sensitivity (SN), positive predictive value (PPV), and alignment time for RNA families that do not contain pseudoknots

	Profile-csHMM								
	***M*-constraint**			**Original**			**Proposed**		
	**SN (%)**	**PPV (%)**	**Time (sec)**	**SN (%)**	**PPV (%)**	**Time (sec)**	**SN (%)**	**PPV (%)**	**Time (sec)**

Transfer RNA	94.2	95.8	0.0739	94.1	96.0	0.0139	93.6	96.2	0.0108
5S ribosomal RNA	94.8	96.3	0.0676	95.1	97.0	0.0024	95.9	98.5	0.0010

In order to test the performance of the proposed method on RNA pseudoknots, we carried out similar experiments using four pseudoknotted RNA families, Corona_pk3, HDV_ribozyme, Tombus_3_IV and Flavi_pk3. For these experiments, we used  and  for all four RNA families. In addition to evaluating the performance of the profile-csHMM method for these families, we evaluated the performance of the PSTAG-based method [20] for comparison. The PSTAG-based structural alignment method is a state-of-the-art pairwise RNA alignment method that uses *pair stochastic tree adjoining grammars (PSTAGs)*. PSTAGs can be used for aligning many known pseudoknots, though not all of them. To the best of our knowledge, the PSTAG-based alignment method [20] is the only grammar-based method that can be used for finding the structural alignment of pseudoknotted RNAs, except for the profile-csHMM method. Table 4 shows the average sensitivity and positive predictive value of the different alignment methods. The sensitivity and the positive predictive value of the PSTAG-based method have been obtained from [20] based on the same test set. As we can see in this table, all four methods could achieve high sensitivity and PPV for the Corona_pk3, HDV_ribozyme, and Tombus_3_IV RNA families. The Flavi_pk3 RNAs could not be aligned using PSTAGs, as they have a more complex secondary structure compared to other RNA families. Unlike PSTAGs, profile-csHMMs can handle RNAs with any kind of base pairs, hence they could be used for aligning Flavi_pk3 RNAs as well. The current implementation can handle any RNAs in the Rivas and Eddy class [26], which includes nearly all known pseudoknots. We can also handle the RNAs outside the Rivas and Eddy class by incorporating additional *adjoining rules*. See [19] for further discussions on adjoining rules and the descriptive capability of profile-csHMMs. Table 4 shows that all three profile-csHMM-based approaches yielded accurate alignment results for Flavi_pk3 RNAs. By comparing the performance of the profile-csHMM method with different constraints, we can note that incorporating the proposed alignment constraint virtually did not affect the alignment accuracy.

**Table 4:** Average sensitivity (SN) and positive predictive value (PPV) for RNA families with pseudoknots

	Profile-csHMM							
	***M*-constraint**		**Original**		**Proposed**		**PSTAG**	
	**SN (%)**	**PPV (%)**	**SN (%)**	**PPV (%)**	**SN (%)**	**PPV (%)**	**SN (%)**	**PPV (%)**

Corona_pk3	95.5	95.7	95.7	96.5	94.8	96.0	94.6	95.5
HDV_ribozyme	94.5	95.1	94.5	95.3	94.2	95.9	94.1	95.6
Tombus_3_IV	95.9	96.4	95.9	96.4	96.8	97.4	97.4	97.4
Flavi_pk3	94.6	96.5	94.5	96.4	94.5	96.8	N/A	N/A

As we can see in Table 5, the proposed sequence alignment constraint was able to significantly improve the alignment speed also for pseudoknotted RNAs. In fact, by comparing the results in Tables 3 and 5, we can observe that the overall computational gain becomes even larger for RNAs with more complicated secondary structures. The new constraint made the alignment speed around 40  100 times faster compared to the fixed *M*-constraint (using ), and around 3  11 times faster compared to the original implementation [17], at a comparable prediction accuracy. We can also note that the PSTAG-based alignment takes considerably longer than the profile-csHMM-based alignment. The large difference in alignment speed is mainly due to the fact that the PSTAG algorithm [20] does not incorporate any constraint to restrict the alignment space.

**Table 5:** Average CPU time for finding the structural alignment of RNAs containing pseudoknots

	Profile-csHMM			PSTAG
	***M*-constraint**	**Original**	**Proposed**	
	**Time (sec)**	**Time (sec)**	**Time (sec)**	**Time (sec)**

Corona_pk3	9.37	0.71	0.23	19.65
HDV_ribozyme	10.30	1.03	0.13	158.77
Tombus_3_IV	6.99	0.35	0.07	193.06
Flavi_pk3	13.31	3.96	0.35	N/A

It would be also interesting to see how the parameters used for predicting the alignment constraint would affect the overall performance. For this purpose, we repeated the previous experiment using different values of  and . Three pairs of  were chosen based on the experimental results shown in Figure 7, such that the average misalignment probability does not exceed 5% for both tRNAs and 5S rRNAs. We used  in all three cases, such that the minimum length of the seed region is one. Note that if there are regions with more than  consecutive matches in the sequence-based alignment, the lengths of the corresponding seed regions will be longer than this minimum. In all three experiments, the parameter  was set to zero. Table 6 shows the sensitivity, PPV, and alignment time for different pairs of . In general, small  and  tend to increase the fraction of bases included in the seed regions, thereby reducing the overall alignment space. As a consequence, the alignment time becomes smaller as we can see in Table 6. However, if these values are made too small, the resulting alignment space can become too restricted, hence degrading the alignment accuracy. This phenomenon could be observed when aligning the Corona_pk3 RNAs with  and . 

**Table 6:** Performance of the proposed approach for different parameters

	Profile-csHMM (Proposed)								
	****			****			****		
	**SN (%)**	**PPV (%)**	**Time (sec)**	**SN (%)**	**PPV (%)**	**Time (sec)**	**SN (%)**	**PPV (%)**	**Time (sec)**

Corona_pk3	92.9	94.4	0.139	94.8	96.0	0.232	95.3	96.2	0.278
HDV_ribozyme	93.2	95.7	0.131	94.2	95.9	0.133	94.5	95.5	0.147
Tombus_3_IV	96.5	97.1	0.065	96.8	97.4	0.068	96.6	97.3	0.069
Flavi_pk3	94.8	97.2	0.329	94.5	96.8	0.351	94.5	96.8	0.362

In Tables 3 and 5, we have shown that the proposed alignment constraint can significantly reduce the average computational requirement for finding the RNA structural alignments. Since the proposed method estimates the constraint based on the sequence alignment of the given RNAs, the actual reduction in complexity will depend on the degree of sequence similarity between the RNAs. Suppose we have a reference RNA of length  and a target RNA of length . In the best case, when these RNAs are perfectly aligned, the overall computational cost will be dominated by the constraint estimation step, hence the resulting complexity will be . In the worst case, the complexity will be identical to that of an unconstrained profile-csHMM alignment, which is  for RNAs without pseudoknots,  for typical RNA pseudoknots (including Corona_pk3, HDV_ribozyme, Tombus_3_IV, and Flavi_pk3 used in our experiments), and  for RNAs with the most complicated secondary structure in the Rivas and Eddy work [26]. In general, the maximum distance between the alignable bases will be limited by the constraint (12). If we define  as(18)

the computational complexity of the profile-csHMM alignment method with the proposed constraint will be  for RNAs that do not contain pseudoknots. For pseudoknotted RNAs in the Rivas and Eddy work, the complexity will range between  and .

## 5. Concluding Remarks

In this paper, we proposed a new method for finding an effective alignment constraint for fast and accurate structural alignment of RNAs. The proposed method is especially useful for accelerating the dynamic programming alignment algorithm of family-specific models, such as the profile-csHMMs or CMs. The alignment constraint proposed in this paper is not very sensitive to small parameter changes in the model that is used to predict the constraint. Therefore, it can be especially useful when we do not have enough sequences in the reference RNA family for training the model. We applied the new constraint to the profile-csHMM-based structural alignment method [17], and evaluated its performance using several RNA families containing pseudoknots. Experimental results showed that the proposed alignment constraint could significantly reduce the alignment time without any loss of alignment accuracy. Although we have mainly focused on incorporating the proposed constraint into the profile-csHMM-based method, these constraints can certainly be used to expedite other alignment methods based on CMs [3, 23] or PSTAGs [20].
